# Challenges influencing nurse-initiated management of antiretroviral therapy training and implementation in Ngaka Modiri Molema district, North West province

**DOI:** 10.4102/hsag.v25i0.1174

**Published:** 2020-03-16

**Authors:** Sheillah H. Mboweni, Lufuno Makhado

**Affiliations:** 1 Aurum institute, Johannesburg, South Africa; 2 Department of Public Health, School of Health Science, University of Venda, Thohoyandou, South Africa

**Keywords:** HIV programme, NIMART implementation, NIMART training, PHC facilities, professional nurses

## Abstract

**Background:**

The increasing number of people testing human immunodeficiency virus positive and who demand antiretroviral therapy (ART) prompted the Department of Health to adopt World Health Organization’s task shifting where professional nurses (PNs) initiate ART rather than doctors. This resulted in decentralisation of services to primary healthcare (PHC), generating a need to capacitate PNs on nurse-initiated-management of ART (NIMART). The impact of NIMART was assessed and even though there was an increased number of patients on ART, the quality of care is of serious concern.

**Aim:**

The aim of this study was to explore and describe the challenges influencing NIMART training and implementation amongst PNs and programme managers.

**Setting:**

The study was conducted from the PHC facilities, in the rural districts of the North West province.

**Methods:**

An exploratory programme evaluation and contextual research design was used in the study. Purposive sampling was used. Focus group discussion (*n* = 28) and individual interviews were used to collect data. Data was analysed using ATLAS.ti software.

**Results:**

The results revealed two themes: inadequacy in NIMART training and the healthcare system challenges that influence NIMART training and implementation. Theme 1 included among others the lack of standardised curriculum and model or conceptual framework to strengthen NIMART training. And theme 2 included patient and district healthcare structural system.

**Conclusion:**

There a need to improve NIMART training and implementation through the standardisation of NIMART curriculum, introduction of pre-service NIMART training in institutions of higher learning, addressing staff shortages and negative attitude of PNs providing ART.

## Introduction and background

The increasing number of people living with human immunodeficiency virus (HIV) and the increasing demand for antiretroviral therapy (ART) exert increased pressure on the South African healthcare system which is already experiencing challenges in human resources (HRs) (Joint United Nations on HIV/AIDS [UNAIDS] 2017). The availability of skilled healthcare providers in South Africa is also a scarcity, especially in rural primary healthcare (PHC) facilities who should provide high-quality human immune virus or acquired immune deficiency syndrome, sexually transmitted infections and tuberculosis (HAST) services in the PHC facilities (Ousman et al. 2016; Sifanelo 2010; World Health Organization [WHO] 2007). According to Simelela and Venter (2014), the Republic of South Africa (RSA) adopted WHO recommendations of task shifting where professional nurses (PNs) initiate ART to complement doctors and address the challenge of inaccessibility to ART services from the PHC facilities. This task shifting calls for the intense training of PNs on nurse-initiated management of antiretroviral therapy (NIMART) in PHC facilities in the country. Although there is a slight decline of 0.8% from 2000 in the global HIV prevalence rate, according to WHO (2012), the prevalence is still high at 19.1%. Only 37% of adults and 24% of children in the world living with HIV receive ART (UNAIDS 2014). Consequently, the prevalence of HIV and tuberculosis (TB) co-infected cases is also increasing, adding to the burden in the management of both cases (UNAIDS 2014). In 2012, there were 9.6 million new TB cases of which 1.2 million were amongst people living with HIV (PLWH) globally (WHO 2012). In sub-Saharan Africa, 45% of PLWH know their HIV status. Those receiving ART were 39%, while those with suppressed viral load (VL) were 29% (UNAIDS 2014). These statistics are raising a concern regarding HIV management worldwide. In South Africa, HIV prevalence amongst the general population is estimated to be 13.5%, with only 4.4 million initiated on ART (Statistic South Africa 2019). There is a noticeable decline amongst children born with HIV because of the successful implementation of the prevention of mother-to-child transmission (PMTCT) programme, reducing the mortality rate by 20% (UNAIDS 2017). According to Statistic South Africa (2019), the life expectancy at birth in the country is still below the target of 70 years, with females estimated at 67.7 years and males at 61.5 years, while HIV-related deaths were rated fifth at 21 830, thus 4.8% of total deaths. The incidence of pulmonary TB in PLWH infection is also increasing nationally, and specifically in the rural districts of the North West (NW) province. The regional training centres (RTCs) were established in all nine provinces of RSA to capacitate at least 75% of PNs in each PHC facility with the necessary knowledge, skills and confidence to initiate ART (North West Province Department of Health 2015–2016). However, gaps still exist in terms of quality of training and implementation in the PHC facilities. The researcher has observed gaps where PNs do not implement what they have been trained to, or do not comply with changes of the National Department of Health (DOH) policies and guidelines. Often, they neither record nor report accurately. In other instances, they are not mentored efficiently or effectively to become competent to initiate and manage ART. Another gap is poor management of data on ART. These need to be investigated to make recommendations that could strengthen NIMART training and implementation.

The Ngaka Modiri Molema (NMM) district in the NW province has not achieved the expected outcomes of the implementation of the NIMART programme as mandated by the WHO 90-90-90 strategy, with only 76% of patients who tested HIV-positive being initiated on ART, which is far below the target of 90%, and the performance fluctuating monthly (North West Province Department of Health, 2015–2016), despite 75% of PNs in PHC facilities having been capacitated on NIMART by the RTC (North West Province Department of Health, 2015–2016). The lack of achievement of the expected outcomes was observed during the initial monitoring and evaluation of the impact of NIMART training on the HIV programme conducted by NMM RTC, where there was fluctuation on new ART initiations, especially paediatrics, TB or HIV co-infected and antenatal care (ANC) pregnant women (North West Province Department of Health, 2012–2015). In addition, according to the NMM, HIV Programme Report (2016), there is a high loss to follow-up (LTFU) compounded with fluctuating initiation rates, leading to a low number of total patients remaining on ART (TROA). The LTFU is estimated at 19%, that is, 14% above the target of 5%, which suggests challenges with adherence and retention of patients. A challenge with the implementation of NIMART in the PHC facilities includes poor collection of blood to monitor the VL which is currently estimated at 54% instead of the benchmark of 90%. Viral load measurement is used to monitor the effectiveness of ART in lowering VL suppression. From the sample used during the initial study, it has been established that 56% of patients on ART show an unsuppressed VL, a figure far below the target of 90% of patients on ART (North West Province Department of Health 2015–2016; North West Province Department of Health, 2012–2015). The aforementioned elements led the researcher to pose the following research question: what are the challenges influencing NIMART training and implementation on HIV and TB management in the PHC facilities of NMM district?

The purpose of the study was to explore and describe the challenges influencing NIMART training and implementation on HIV and TB management to identify the gaps and make recommendations that might improve the quality of care for PLWH in the NMM district, NW province.

## Material and methods

### Research design

An exploratory programme evaluation and contextual research design was used in the study to explore and describe challenges influencing NIMART training and implementation amongst PNs and programme managers.

### Context of the study

The study was conducted in the rural PHC facilities of the NMM district of NW province. The province has four districts and has decentralised the HIV and TB programme to the PHC level to increase access to ART and comply with the implementation of district healthcare system policies. The district regional and tertiary hospitals are used as referral points for complicated cases in need of specialised care. The NMM district has five sub-districts, with 94 PHC facilities, and participants were working under the five sub-districts.

### Population and sampling

The population of the study include all 447 (94%) NMM district PNs trained on NIMART and HIV programme managers. The NMM district had a total of 476 PNs even though the district was experiencing high staff turnover monthly (North West Province Department of health, 2015–2016).

**Inclusion criteria:** Professional nurses were trained on the implementation of NIMART from PHC facilities and managers were directly involved in the implementation and management of the HIV or TB programme.

**Exclusion criteria:** Professional nurses were not trained on NIMART and managers were not directly involved in HIV or TB management.

A non-probability purposive sampling method was used in the study. Participants who meet the inclusion criteria were recruited to participate voluntarily through the letter written to the sub-district and cluster managers, with personal, academic and contact details of the researcher. The list of those who accepted to participate was sent back by e-mail (Babbie & Mouton 2011).

## Data collection methods

A focus group discussion (FGD) was conducted to collect data from PNs trained on NIMART and individual interviews were held with three programme managers.. Each sub-district has one HIV and TB programme manager and only three volunteered to participate; however, data saturation was reached with this sample. The main question asked in both the FGDs and individual interview was as follows: what are the challenges influencing NIMART training and implementation in NMM district PHC facilities? This was followed by probing questions regarding the quality of training, mentoring, guideline updates, linkage and retention of PLWH into care. Five FGDs were conducted with 28 participants and data saturation was reached with this sample size. Both interviews were conducted in a private room according to participants’ own convenience. Arrangement was made by the researcher regarding the time to conduct FGDs, which was after work, during lunch time or after cluster meetings to avoid interruption of services. The interviews were digitally audio-recorded.

Ethical clearance to conduct the study was obtained from the NW University research committee. Gatekeeper permission was obtained from the Provincial Department of Health (DOH; Ref no. NW 201711006), district, sub-district, cluster and facility managers. Written consent was obtained from each participant before participating in the study and each FGD or individual interview lasted for 90–120 min, with FGDs consisting of 5–7 participants at a time, and the interview was conducted in English. Participants, however, sometimes used Setswana while responding, which was interpreted in English by the researcher (De Vos et al. 2012). Demographic or personal data of the participants were collected through a questionnaire attached to the consent form before conducting the interview (see Table 1) in order to have a better understanding of the experience, position and whether they were assessed for NIMART competency or received mentoring to make sound conclusion of the study. Numbers were used instead of names to ensure confidentiality and anonymity of the participants.

**TABLE 1:** Demographic or personal data of participants (*n* = 28).

Demographic	Characteristics	Frequency	Percentage
Gender	Male	2	7
	Female	26	93
Age	20–30 years	3	11
	31–40 years	6	21
	41–50 years	15	54
	50 years and above	4	14
Position	Clinical nurse	20	71
	Clinical practitioner	5	18
	HIV programme manager	3	11
Experience	1–5 years	11	39
	6–10 years	7	25
	11–15 years	4	14
	16–20 years	2	7
	21–25 years	2	7
	26 years and above	2	7
Qualification	Diploma	14	50
	Post basic diploma	6	21
	Degree	8	29
Geographic area	Rural	22	79
	Urban	2	7
	Semi-rural	4	14
Type of PHC facility	Clinic	19	68
	Community Health Centre	6	21
	Sub-district office	3	11
NIMART certificated	Yes	17	61
	No	11	39
Initiating ART in PHC facility	Yes	23	82
	No	5	18
Mentoring others in PHC facility	Yes	9	32
	No	19	-

HIV, human immunodeficiency virus; ART, antiretroviral therapy; NIMART, nurse-initiated management of antiretroviral therapy; PHC, primary healthcare.

### Data analysis

A descriptive qualitative data analysis was used in the study. Data analysis occurred simultaneously with data collection (Grove et al. 2013). The demographic data were captured in a Microsoft Excel spreadsheet and descriptive statistics were used to summarise this information in tables. The audio-recorded data were transcribed verbatim. The ATLAS.ti program was used to analyse the qualitative data supplemented by the basic steps of Notice-Collect Think (Friese 2019). The Notice-Collect Think was conducted in two phases or levels: the descriptive and conceptualising analysis. During the descriptive phase, the researcher read and re-read the transcripts and field notes and then identified patterns of the data and started coding and verifying codes. In the conceptualisation phase, the ATLAS.ti program was used to link and classify similar data together into categories and themes (Friese 2019). Two main themes emerged as shown in Table 2.

**TABLE 2:** Themes, categories and sub-categories of the nurse-initiated management of antiretroviral therapy training and implementation challenges.

Number	Theme	Categories	Sub-categories
1	NIMART training challenges	1.1. Lack of NIMART standardised curriculum	1.1.1. Poor integration of theory and practice
			1.1.2. Inadequate facilitation skills
		1.2. Lack of involvement of training, education and practice stakeholders in NIMART training and implementation	1.2.1. Lack of involvement of quality assurance bodies, nursing colleges and universities
			1.2.2. Lack of pre-service training
		1.3. Inadequate continuous professional development	1.3.1. Inadequate continuous in-service
		1.4. Lack of training model or conceptual framework	1.4.1. Lack of guidance in NIMART training and implementation
2	Healthcare system challenges	2.1. Patient-related challenges	2.1.1. Poor socio-economic status and adherence to treatment
			2.1.2. Lack of disclosure
		2.2. Human resource challenges	2.2.1. Shortage of skilled healthcare professionals
			2.2.2. Staff attitude on HIV management
			2.2.3. Lack of confidence to manage children and other complicated cases
		2.3. Poor data management	2.3.1. Poor adherence to data management SOPs
			2.3.2. Lack of data verification
			2.3.3. Incomplete clinical records
			2.3.4. Inadequate clinical records quality audits
		2.4. Poor infrastructure	2.4.1. Poorly maintained PHC facilities
			2.4.2. Overcrowding
		2.5. Inadequate district healthcare structural systems	2.5.1. Lack of district mentoring strategy
			2.5.2. Lack of support systems from management
			2.5.3. Poor compliance to policies and guidelines

HIV, human immunodeficiency virus; NIMART, nurse-initiated management of antiretroviral therapy; PHC, primary healthcare; SOP, standard operating procedure.

### Trustworthiness of the study

Trustworthiness in qualitative research is achieved by enhancing credibility, dependability, confirmability and transferability (Polit & Beck 2017).

The credibility of the study was enhanced by spending more time with participants in FGDs as well as individual interviews for about 90–120 min until data saturation was reached. The audio-recorded interviews were transcribed and interpretations were shared with the participants to validate if their experiences were competently and accurately captured. Dependability was enhanced by maintaining an audit trail through keeping all copies of notes, transcribed and recorded data for future use, including supplying participants with researchers’ personal and academic information for contact or explanation at any time.

Confirmability was enhanced by conducting a pilot study, which served as a pre-test to the interview schedule, and interviewing skills from six PNs trained on NIMART from another district in the NW province. The results of the pilot are not a part of the report presented in the final study. Conducting of a pilot study in another district with NIMART-trained nurses helped in refining the study methods. An independent co-coder was used, and three consecutive discussions followed before consensus was reached on the study methods and phrasing of the research question. Transferability was enhanced using non-probability purposive sampling methods to collect data from PNs trained on NIMART and managing the HIV and TB programme.

### Ethical consideration

Ethical approval to conduct the study was obtained from the North-West University (NWU) ethics committee and permission to collect the data was obtained from the NW province. Participation was voluntary and all the participants were informed of the right to withdraw at any stage of the study. Anonymity and confidentiality of participants were maintained by not using their names in the demographic questionnaire during the FGDs. Participants were informed that interviews will be tape-recorded and they needed to sign consent forms. The master list was kept separately and under lock and key from the questionnaire, consent forms and tape recorder.

## Results

### Demographic data of participants

Five FGDs were conducted with 28 PNs trained on NIMART from NMM PHC facilities who participated in the study. Table 1 presents the summary of the participants’ personal or demographic data and reveals that more PNs (89%) that provided direct nursing care participated in the study and that only three PNs (11%) are programme managers. The majority of participants were females (93%), which suggest that nursing is dominated by women, who are said to be caring in nature. The majority of participants (54%) were aged 41–50 years; 39% of the participants had a work experience of 1–5 years and 50% of the participants had a basic diploma in nursing. The majority of participants were working in rural PHC facilities (79%) and 68% of PNs were working at PHC clinics. Although all the participants were trained on NIMART, only 61% received mentoring after NIMART training, assessed and certificated for NIMART competence and 82% reported to be initiating ART in their facility, while 18% were no longer providing direct patient care but were managing the HIV and TB programme. Amongst the PNs who participated, 32% were mentoring others in the PHC facility on NIMART implementations voluntarily, as they have the ability and willingness to transfer skills and knowledge to others.

### Challenges influencing nurse-initiated management of antiretroviral therapy training and implementation as perceived by professional nurses and managers

Table 2 presents a summary of the challenges experienced by NIMART-trained PNs and programme managers in the implementation of NIMART training. These challenges bear a negative impact on the training and implementation of NIMART. Two themes were identified, that is, the NIMART training and healthcare system challenges. The first theme, NIMART training challenges, revealed four categories, which were lack of standard curriculum with two sub-categories, poor integration of theory and practice, and inadequate facilitation skills. The involvement of other stakeholders to improve quality of training includes higher education institution (HEI), Health and Welfare Sector Education and Training Authority (HWSETA) and South African Nursing Council (SANC) implementation of continued professional development through continuous in-service training and lack of conceptual framework or model to guide or strengthen NIMART training and implementation.

The second theme identified is the healthcare system challenges that revealed five categories: patient, HR, poor data management, poor infrastructure and inadequate district health system (DHS) structural systems challenges. The patient challenges identified were patient’s poor socio-economic status, lack of disclosure and poor adherence to treatment and appointments. The HR challenges revealed shortage of skilled healthcare providers in PHC facilities, negative attitude, lack of confidence and inadequate skills to initiate ART. The inadequate DHS structural system category revealed poor compliance to policies and guidelines, lack of district mentoring strategy and support by management from district to cluster level, including programme managers. The poor data management category revealed poor compliance to data management standard operating procedure (SOP), which includes verification and validation, incomplete clinical records and lack of clinical records audit to identify gaps. The last category revealed poor infrastructure which identified small and poorly maintained PHC facilities that are overcrowded by the increasing number of clients in need of ART and other chronic conditions therapy.

### Theme 1: Nurse-initiated management of antiretroviral therapy training challenges

Participants were of the view that there is a lack of standard curriculum, involvement of education, training and practice stakeholders to ensure quality, inadequate continued professional development and mere absence of the conceptual framework or model to guide and strengthen NIMART training and implementation in the district, as well as in the province.

#### Category 1.1. Lack of standardised nurse-initiated management of antiretroviral therapy curriculum

Participants highlighted that NIMART training is partner driven. The RTCs do not have a curriculum developed by the district, province or national DOH. Training is provided and conducted by the US President’s Emergency Plan for AIDS Relief (PEPFAR) partners supporting the DOH in prevention, control and management of HIV and TB programme. The period and content of NIMART training differs; some attended sessions for 3 months, while others attended over 5 or 10 days theory classes.

There further seemed to be a greater focus on theory than on practice, content not aligned with the number of days. One PN revealed that:

‘I was trained for 5 days, with too much content.’ (P7, FGD1, female)

Another one added that:

‘I was trained for 10 days, but there was no practice, only theory.’ (P20, FGD5, female)

Participants emphasised that the process of the final assessment for competency differed between the groups that completed the training. This raises question of competence in HIV and TB management. One PN highlighted that:

‘I completed a test before and after training.’ (P18, FGD4, female)

Another one added that:

‘I only completed POE [*Portfolio of Evidence*].’ (P19, FGD4, male)

**Sub-category 1.1.1. Poor integration of theory and practice:** Participants reported that facilitators put more emphasis on theory than on enhancement of practical skills during NIMART training. Therefore, they find it difficult to grasp the necessary skills, to gain confidence and be competent in implementing the strategies learnt after they had gone through training, while still being without a mentor to support them in the facility, to transfer skills.

Participants had the following views:

‘They talk about clinical stationery and registers but never show us how to complete [*it*].’ (P16, FGD4, male)

‘I expected them to at least take us for practice, maybe two days, one for adults and one for children to initiate treatment like in IMCI [*Integrated Management of Childhood Illness*].’ (P10, FGD3, female)

The participants expressed that they were overwhelmed by the information presented to them with the facilitator rushing to complete the course content, but not paying attention to whether they showed understanding of the information obtained or could apply what they have learnt. Therefore, the translation into practice was poor, as shown in the following quotes:

‘Yoooh [*sic*], the slides were too much, I was so tired.’ (P21, FGD5, female)

‘The trainer was just reading [*and*] rushing to complete, five days was not enough, too much information.’ (P6, FGD2, female)

**Sub-category 1.1.2. Inadequate facilitation skills:** Participants who also trained PNs on NIMART reported that they never received formal general facilitation skills to know how to use various teaching strategies, methods and media to meet the diverse needs of the participants. The PNs verbalised that they were selected only based on experience of managing HIV and not on their competency of facilitation of NIMART. One PN stated that:

‘I just facilitated the content as per orientation; I never go for facilitation skills training.’ (P7, FGD2, female)

Another participant added that:

‘I just have [*a*] passion for HIV management but do not have facilitation skills.’ (P10, FGD2, female)

#### Category 1.2. Lack of involvement of education, training and practice stakeholders in nurse-initiated management of antiretroviral therapy training and implementation

Participants revealed that they were not visited or seen by the quality assurance bodies during training or assessment. They were further of the view that there was a lack of NIMART pre-service training.

**Sub-category 1.2.1. Lack of involvement of quality assurance bodies:** Participants were not sure if NIMART is recognised by the SANC or HWSETA, or supported by nursing colleges and the university’s Department of Nursing. The participants indicated that they never received any certificate from these bodies after training and they have tried to enquire from institutions and nobody knows about NIMART training programme.

One participant explained:

‘I have never seen or heard that SANC, HWSETA or university people visiting us during training or assessment.’ (P4, FGD1, male)

Another one added that: ‘No quality assurance bodies [*are*] involved except the partners funded by PEPFAR.’ (P10, FGD3, female)

**Sub-category 1.2.2. Lack of nurse-initiated management of antiretroviral therapy pre-service training:** Participants expressed their frustration in not knowing how to initiate ART. When starting employment, after completion of training from a college or university, they continued to turn patients away from the healthcare facility and advised them to come back another day – an action which contributed to the delay in ART initiation from their facility when their colleagues who are NIMART trained are not on duty. A PN expressed that:

‘I was so disappointed and frustrated at the same time for failing to help the client.’ (Emotional, raising the voice) (P8, FGD2, male)

In addition, another PN expressed that:

‘[*I*] wish NIMART can be introduced in the college or university curriculum so that I know how to manage TB/HIV patients before I join the department [*of health*].’ (P13, FGD3, female)

#### Category 1.3. Inadequate continued professional development

Participants revealed that NIMART is not yet considered as part of Continuing Professional Development (CPD) as SANC has not yet finalised the CPD point for nurses.

**Sub-category 1.3.1. Inadequate continuous in-service training:** Participants indicated that there is inadequate in-service training on the changes in guidelines, policies and protocols, which results in poor compliance and inefficiency in the implementation of NIMART; there are times during which they only receive guidelines in the facilities, without any explanation, resulting in misinterpretation. This view that there is inadequate in-service training was expressed by a participant as follows:

‘Eeeish! I learn about the new guidelines when I have done something wrong during patient management and feel bad.’ (Feeling sad and emotional) (P3, FGD1, female)

Another PN added that:

‘I learn about new changes in guidelines from partners supporting my facility.’ (P2, FGD2, female)

Participants reported that there is a lack of feedback from those who attend in-service training in the facility.

The process of in-service training should provide an update regarding guidelines for NIMART nurses in PHC facilities but failed to cover all who should be implementing it, which might result in the mismanagement of patients, conflict and litigation, in some instances. The view of inadequate in-service training was highlighted by a PN, stating that:

‘Some do attend [*in-service training*] but [*we get*] no feedback.’ (P20, FGD5, female)

Another PN added:

‘Even my supervisor in the facility knows nothing about the new guideline.’ (P23, FGD 5; male)

#### Category 1.4. Lack of training model and conceptual framework

Participants also revealed that there was no conceptual framework or model guiding NIMART training. Each province or district has its own curriculum and ways of conducting training and needs serious attention. This view that there is a lack of training model and conceptual framework was verbalised in the following way:

‘I was not told of any framework or model to follow; I know nothing about that.’ (P22, FGD5, male)

Another participant stated that:

‘The RTC and provincial office did not tell us of any framework.’ (P24, FGD5, female)

### Theme 2: Healthcare system challenges

Participants were of the view that the healthcare system is experiencing patient-related challenges such as poor socio-economic status, adherence to treatment and disclosure of HIV-positive status. Again, poor data management, shortage and negative attitude of staff were viewed as affecting PHC facilities’ performance negatively and discouraging PNs and programme managers. Furthermore, challenges of poor infrastructure, inadequate DHS structure systems which include lack of mentoring strategy and support from management were raised by the participants.

#### Category 2.1. Patient-related challenges

Participants reported that patients’ socio-economic factors play a major role in the adherence to treatment and affect implementation of NIMART and performance of the HIV and TB programme. Lack of disclosure is also viewed as affecting adherence to treatment and honouring of follow-up appointment dates.

**Sub-category 2.1.1. Poor socio-economic factors and adherence to treatment:** Participants reported that the patient plays a major role in the success of the ART programme. Patients’ socio-economic factors, lack of disclosure, not honouring of follow-up appointments and poor adherence to treatment negatively affect management and retention to care. This was observed because of high LTFU and TROA fluctuation. This concern was revealed by the following PNs:

‘Patients are always moving from one place to other, looking for jobs.’ (P7, FGD2, male)

And

‘Patient move to farms and mining areas for work without informing the facility they don’t honour their appointments.’ (P8, FGD2, female)

**Sub-category 2.1.2. Lack of disclosure:** The participants viewed patients’ lack of disclosure of their HIV-positive status to family, friends and partners as a serious challenge in the management of HIV and TB patients. Most patients missed their appointment dates, leading to non-adherence to treatment. This might lead to high LTFU in PHC facilities and drug resistance.

The following statements were recorded from the participants:

‘Children and adolescents are brought late to care or default treatment because their status is unknown to care givers.’ (P2, FGD1, female)

‘During counselling I discovered that the mother did not disclose why the child is taking medication daily.’ (P4, FGD 2, female)

#### Category 2.2. Human resource challenges

**Sub-category 2.2.1. Shortage of skilled healthcare providers:** Participants reported that there is high staff turnover of skilled HRs in HIV and TB management and the remaining staff members are under pressure to deal with the large numbers of PLWH demanding ART. Patients also complain of long waiting period for care. Some participants stated that:

‘I’m the only PNs in the facility, I’m overworked, so exhausted since ART initiation takes time and I have other responsibilities’ (Very sad). (P5, FGD1, female)

‘Some patients leave without treatment.’ (P4, FGD1, female)

**Sub-category 2.2.2. Staff attitude:** Participants indicated that some PNs had a negative attitude towards the management of HIV and TB patients, despite having been trained on NIMART and they often call PNs appointed by partners for initiation. These actions delay linkage to care. Professional nurses highlighted that:

‘Some are being emotional [*when treating patients*], it’s immoral, the client was told to come back, and baseline [*blood tests*] were not taken.’ (P4, FGD1, female)

‘Some lack interest, I wonder why they attend training?.’ (P6, FGD2, female)

**Sub-category 2.2.3. Lack of confidence to manage children and other complicated cases:** Participants revealed that they still lack confidence on managing paediatric cases which raise serious concern as they are also IMCI trained and are provided with a step-by-step guide on how to manage sick infants and children, although some indicated that children are often not presented early enough at the PHC facility. It was stated by PNs that:

‘I am scared to manage infants.’ (P20, FGD5, female)

And that:

‘They [*children*] presented being very ill with complications and I have to transfer to hospital because when tested positive the caregivers or mothers never come back for treatment.’ (P1, FGD1, female)

Participants also revealed that even during completion of portfolios of evidence (POEs) after training, they do not get enough cases of children, pregnant women and adults with HIV and TB co-infection and used scenarios to learn its management. This might contribute to the lack of confidence to manage children and co-infected patients. This is echoed in the following statements:

‘I got few cases for my POEs; the rest I simulated using scenarios.’ (P17, FGD4, female)

‘Paediatric cases are very rare in my facility and I [*have*] forgot[*ten*] [*the*] management.’ (P8, FGD3, male)

#### Category 2.3. Poor data management

Participants expressed that they are working very hard to achieve the set target, but there is so much data related to the factors that hamper performance of the HIV and TB programme, and these variations in challenges discourage NIMART-trained nurses. These factors included poor compliance to the data management SOP, lack of data verification, incomplete clinical records and inadequate clinical record audits.

**Sub-category 2.3.1. Poor compliance to data management standard operating procedures:** Participants reported that operational managers are not complying to data management SOP, as developed by the DOH to improve the quality of data in PHC facilities, sub-district and district levels and reflect badly on their performance. Professional nurses revealed that:

‘Some [*of the*] data is not captured especially TB/HIV and ANC ART initiation eeish data man yare bulaya [*It’s killing us*] [*sic*]. In my facility there is a pile of files that are not captured.’ (P4, FGD1, male)

And that:

‘OPM [*Operational manager*] and information officers are not verifying data.’ (P11, FGD3, female)

**Sub-category 2.3.2. Lack of data verification:** Participants reported that data are not verified; OPMs or supervisors rely on data capturers who also make mistakes or misplace some files that are not yet captured, and it is only a clinical person who can identify the gaps, which is evident from the following statements by some PNs:

‘I am an OPM, I do not perform my duties as most of the time I have to provide direct care due to shortage of staff or other sub-district or district activities.’ (P21, FGD5, female)

‘OPM and information officers do not conduct data verification and validation.’ (P19, FGD4, female)

**Sub-category 2.3.3. Incomplete clinical records:** Participants reported that the increasing number of patients in need of ART and other HIV services add to the pressure on PNs in such a way that they do not have time to complete registers, clinical stationery and record patients’ data appropriately. A clinical stationery is a form designed by the DOH to standardise recording of HIV-positive patient’s information by clinicians and is also used by data team to capture in Tier.net system in South Africa. One PN highlighted that:

‘There is too much of writing, patients complain and put pressure on me, [*I am*] alone in the clinic and I forgot [*forget*] to complete the records later.’ (P4, FGD1, male)

Another participant emphasised that:

‘Data capturers always complain that clinical stationery is not complete.’ (P20, FGD5, female)

**Sub-category 2.3.4. Inadequate clinical record quality audits:** Participants reported that OPMs or supervisors were expected to perform their management role of auditing clinical stationery, registers and patient files to ensure compliance to policies and guidelines, but this is not consistently done because of competing activities, high numbers of patients and shortage of staff, as highlighted by the following participants:

‘As a programme manager, I’m expected to audit records and provide feedback, but I have so many activities that interfere with my programme.’ (P21, FGD5, female)

‘I need extra hands to support me to perform clinical audits and mentoring.’ (P22, FGD5, female)

#### Category 2.4. Poor infrastructure

Participant viewed poor infrastructure as one of the challenges affecting NIMART implementation in the PHC facilities and indicated that there is poor maintenance and overcrowding as the PHC facilities are very small to accommodate the increasing number of patients demanding care.

**Sub-category 2.4.1. Poorly maintained primary healthcare facilities:** Participants reported that their working environment is unpleasant, full of cracks, non-functioning toilets, broken pipes, unfunctional windows, ineffective doors, without water and the consultation rooms not enough to render all PHC services, especially counselling rooms for HIV patients; they claimed that management is doing nothing to improve these elements. This view of poorly maintained infrastructure was highlighted in this way:

‘We only have one consultation room and one counselling room.’ (P16, FGD4, female)

‘The walls are having cracks, old paint. Windows and doors broken, toilets for patients not functioning and there is no water.’ (P15, FGD3, female)

**Sub-category 2.4.2. Overcrowding:** Participants emphasised that the facilities are very small and cannot accommodate the high number of patients coming for HIV and TB and other PHC services. This may result in cross-infection. One PN opined that:

‘The waiting area is very small, patients had to stay outside and sometimes it is very cold.’ (P18, FGD4, female)

Another PN indicated that:

‘The facility is always full and congested with patients daily.’ (P19, FGD4, female)

#### Category 2.5. Inadequate district healthcare structural system

Participants revealed that the district healthcare system does not have a system in place to ensure quality of the NIMART training and implementation, and they rely on partners supporting the HIV and TB programme. Professional nurses and programme managers stated that:

‘There is no system in place to strengthen NIMART training and implementation in the district, we rely on partners.’ (P16, FGD3, female )

‘No system in place, I wonder what the DCST [*District clinical specialist team*] is doing, Ooohh!!’ (P19, FGD4, female )

**Sub-category 2.5.1. Lack of mentorship strategy in the district:** Programme managers and PNs revealed that NIMART mentorship is partner driven. Partners refer to PEPFAR-funded organisations that provide health system strengthening on HIV and TB programme in the district. Even though PNs in some departments were trained on clinical mentorship, they were not implementing the strategies they had acquired. One PN had this to say:

‘I’m a mentor but I can’t [*do it*], I have other responsibilities in the facility, there is shortage of staff.’ (P16, FGD3, female)

In addition, PNs highlighted that:

‘There is no mentoring plan in our district, I’m a programme manager with 14 programmes, I’m overworked I need someone to assist in supporting and mentoring facilities.’ (P20, FGD5, female)

Participants highlighted to have received different mentoring strategies from different partners as evident in the claims that follow. This was revealed by one PN:

‘My mentor visits the facility once a week and I sometimes call.’ (P2, FGD1, female)

Another PN shared as follows:

‘I’m supported by my colleagues through WhatsApp or Facebook group [*without mentioning the name of the patient*].’ (P16, FGD3, female)

**Sub-category 2.5.2. Inadequate support from management:** Participants reported that they have not received adequate support from the programme, cluster and district managers regarding challenges related to ART and TB management after training. One participant revealed that:

‘I relied on mentors from partners, colleagues and sometimes from the facility manager, although busy with other activities.’ (P16, FGD3, female)

Another PN stated that:

‘I [*have*] never seen the programme manager coming to mentor or support me after training.’ (P5, FGD1, female)

And another one added:

‘Managers only come when there is a problem or visit by provincial or national Department of Health.’ (P10, FGD3, male)

**Sub-category 2.5.3. Poor compliance to policies and guidelines:** Participants reported that most PNs in the facility are trained on NIMART, but some still do not practise what they have been taught despite mentoring and that non-compliance matters compromise the quality of patient care. This was reported in this way:

‘I do not know; some do not follow guidelines even when they are available in the facility.’ (P3, FGD1, female)

‘Some PNs has [*have a*] negative attitude, they don’t provide counselling just issue treatment.’ (P7, FGD2, female)

## Discussion

The study findings reveal that inadequate training strategies and healthcare systems challenges cumulatively have a negative influence on the implementation of the NIMART training. The RTC is responsible for training PNs on NIMART and is expected to produce knowledgeable, skilled and confident nurses to manage HIV and TB patients; however, the study revealed the use of ineffective training strategies, partner-driven mentoring and training with no standardised curriculum and unskilled facilitators. The revelation has a negative impact on the outcomes of learning and practice in terms of developing critical thinking skills amongst PNs to make sound and ethical decisions in caring and implementing guidelines. Kaposhi, Mqoqi and Schnopflocker (2014) and Byakika-kibwika et al. (2015) confirmed that lack of interactive training, which promotes critical thinking and mentoring, influences implementation. Lack of pre-service NIMART or HIV-training of student nurses had negative impact on implementation and rendering of quality because of knowledge and skills gaps and might lead to stress and frustration in failing to manage HIV and TB patients. This was confirmed by Lekhuleni, Kola and Moomba (2015) that student nurses have insufficient knowledge regarding ART management. Policies and guidelines change frequently and lack of in-service training on the updates and changes leads to improper implementation and poor patient care (Mathibe et al. 2015). Several studies have confirmed that continuous facility-based training is necessary to keep clinicians updated (Oladele et al. 2017; Owens & Moroney 2015). Lack of an appropriate mentoring strategy and relying only on partners affect the transfer of knowledge into practice, confidence and skills. Studies conducted by Smith et al. (2016) and Kufa et al. (2014) confirmed that nurses still lack confidence and skills after training. Dependency on partners might result in serious collapse in the performance of the HIV and TB programme when partners leave the district or facilities. Critical issues of shortage of staff contributed to negative attitudes demonstrated by the staff members (Spies et al. 2016). This stress can be related to the exhaustion from workload and pressure from patients in the overcrowded facilities. In addition, patients experience long waiting hours because of shortage of skilled healthcare providers. Poor infrastructure and overcrowding should be dealt with decisively as it might expose patients and staff members to infection (Davies, Homfray & Venables 2013; Kaposhi et al. 2014; Mack et al. 2015; Mbonye et al. 2016; Spies et al. 2016; Zuber et al. 2014). Overcrowding and unpleasant environment in PHC facilities might also contribute to patients’ poor adherence to appointments for ART initiation, counselling and disclosure to partners as there is not enough time and space for quality individual counselling by PNs, and this was supported by Plazy et al. (2015), even though this can differ amongst men and women. All these factors contribute to high LTFU, fluctuating total number of patients remaining on ART and poor monitoring of antiretroviral’s (ARV) effectiveness through collection of VL, and can eventually lead to complications and death, thus reducing life expectancy. A study conducted in Kenya and Canada on the relationship of adherence to clinic appointment confirmed this (Kimeu et al. 2016; Patterson et al. 2015). Several studies reviewed have revealed similar challenges; however, in this study, the shortage of treatment and equipment was not raised. The lack of management to support the HIV and TB programme affected its performance and this was supported by Ndubuka et al. (2016) that clinical role model is necessary to improve implementation.

## Practical implications

With reference to the study results and recommendations, improvement of NIMART training and implementation could have a positive impact on ART management and monitoring to achieve better programme outcomes and improve the quality of life of PLWH. The recommendations made might also practically improve integration of theory and practice and dealing decisively with challenges affecting implementation in the PHC facilities.

## Limitations of the study

The study only focused on the challenges affecting the NIMART training and implementation with specific reference to the HIV and TB programme. The challenges are only from the perspective of the nurses; all other stakeholders would need to give their inputs when the programme is evaluated to highlight the challenges comprehensively. However, strategies used to deal with barriers for the HIV programme can also be transferred to improve other programmes to render a comprehensive integrated quality care. It was also conducted on a small sample size and in one area only.

## Conclusion

Significant challenges influencing the quality of NIMART training and implementation were revealed. These include training strategies, healthcare system and patient factors, which need to be dealt with to achieve better patient and HIV and TB programme outcomes. However, NIMART training and implementation resulted in the increased access to care in terms of expansion of ART programme by PNs in NMM district PHC facilities.

## Recommendations

Based on the study results, the following is recommended: the RTC; provincial and national DOH, nursing departments from institutions of higher learning; SANC; developmental partners; and other relevant stakeholders should adopt a standardised NIMART training curriculum. The RTC should employ skilled facilitators who will use interactive training strategies to stimulate critical thinking to make sound ethical decision-making in the management of HIV and TB patients. The facilitators should improve clinical practical training to integrate theory and practice. The DOH should mandate Higher education institutions (HEI) offering nursing courses to integrate NIMART in the curriculum to prepare students nurses to render quality HIV and TB care when entering the healthcare system. Programme managers should provide support to PNs trained on NIMART by dealing with barriers that hinder implementation and through development and implementing district mentoring strategy and coaching. The data management team and OPMs should monitor compliance to data management SOPs and conduct weekly data verification, validation and auditing of clinical stationery and registers to improve data quality. The district management team including OPMs should develop a plan to deal with shortage of resources, human and infrastructure. The district management team and OPM also should implement strategies to manage PNs trained on NIMART but not complying with policies and guidelines. Further studies are necessary to develop a conceptual framework or model that could provide guidance and strengthen NIMART training and implementation.
